# Uptake of the glycosphingolipid sulfatide in the gastrointestinal tract and pancreas *in vivo *and in isolated islets of Langerhans

**DOI:** 10.1186/1476-511X-5-26

**Published:** 2006-10-17

**Authors:** Maria Blomqvist, Thomas Osterbye, Jan-Eric Månsson, Karsten Buschard, Pam Fredman

**Affiliations:** 1 Institute of Neuroscience and Physiology, Psychiatry and Neurochemistry Section, The Sahlgrenska Academy at Göteborg University, Sahlgrenska University Hospital/Mölndal, SE-431 80 Mölndal, Sweden; 2 Bartholin Instituttet, Rigshospitalet, DK-2100 Copenhagen, Denmark

## Abstract

**Background:**

The glycosphingolipid sulfatide has previously been found in several mammalian tissues, but information on the uptake of exogenously administered sulfatide in different organs *in vivo *is limited. In pancreatic beta cells, sulfatide has been shown to be involved in insulin processing and secretion *in vitro*. In this study, we examined the uptake of exogenously administered sulfatide and its distribution to the pancreatic beta cells. This might encourage future studies of the function(s) of sulfatide in beta cell physiology *in vivo*. Radioactive sulfatide was given orally to mice whereafter the uptake of sulfatide in the gastrointestinal tract and subsequent delivery to the pancreas was examined. Sulfatide uptake in pancreas was also studied *in vivo *by i.p. administration of radioactive sulfatide in mice, and *in vitro *in isolated rat islets. Isolated tissue/islets were analysed by scintillation counting, autoradiography and thin-layer chromatography-ELISA.

**Results:**

Sulfatide was taken up in the gastrointestinal tract for degradation or further transport to other organs. A selective uptake of short chain and/or hydroxylated sulfatide fatty acid isoforms was observed in the small intestine. Exogenously administered sulfatide was found in pancreas after i.p, but not after oral administration. The *in vitro *studies in isolated rat islets support that sulfatide, independently of its fatty acid length, is endocytosed and metabolised by pancreatic islets.

**Conclusion:**

Our study supports a selective uptake and/or preservation of sulfatide in the gastrointestinal tract after oral administration and with emphasises on pancreatic sulfatide uptake, i.p. administration results in sulfatide at relevant location.

## Background

Sulfatide (3'-sulfo-galactosylceramide) is a glycosphingolipid which has previously been found in substantial amounts in the nervous system, kidneys, gastrointestinal tract, liver and within the insulin producing beta cells in pancreas [1-3]. Several functions have been attributed to sulfatide, such as interaction with proteins in the extracellular milieu, involvement in bacterial-host binding, interaction with membrane component such as receptors and ion channels [1] and regulation of immunological cells [4-9]. Studies during the past decade also suggest that sulfatide is involved in insulin processing and regulation of insulin secretion *in vitro *[10-12] and is thus a molecule to be considered in the context of beta cell function.

The sulfatide fatty acid isoform varies between cell types and tissue structures. In the brain, which has a high sulfatide content, the major source is myelin where sulfatide mainly contains long unsaturated and hydroxylated fatty acids. In the pancreatic beta cells, saturated fatty acids without hydroxylation are major isoforms. The short 16 carbon atom (C16:0) isoform comprises one and the longer 24 carbon atom (C24:0) the other major species in islets [10,13]. The length, saturation and hydroxylation of the fatty acid chain is one of the factors affecting the physical/chemical properties of the molecule, and several studies suggest that the fatty acid composition of glycosphingolipids is important for its biological function(s) [14-17]. Our results suggest that this is also the case for pancreatic beta cells. Among the two major isoforms of sulfatide in beta cells, the short chain isoform, C16:0, is responsible for insulin crystal preservation *in vitro *[3].

Type 2 diabetes patients are characterised by insulin resistance and impaired insulin secretion, and animal models are commonly used for pathophysiological studies of this disease. The beta cell related C16:0 sulfatide isoform was found in reduced amounts in the type 2 diabetes animal models ob/ob and db/db mice, as compared with non-diabetic Lewis rats, BALB/c mice and human pancreatic tissue [3]. Furthermore, intra peritoneal administration of C16:0 sulfatide to pre-diabetic Zucker rats resulted in an ameliorated glucose induced insulin secretion [18]. This was associated with an increase in the pancreatic sulfatide content, but it was not determined whether this increase was due to an uptake of sulfatide in the beta cells. Further support for the involvement of C16:0 sulfatide in insulin secretion includes recent results [19], derived from patch clamp experiments, where this specific isoform was shown to be involved in the regulation of insulin secretion as described previously [12], where a mixture of sulfatide isoforms was used.

In anticipation of further studies of the role of sulfatide in beta cell physiology *in vivo *and its potential pharmacological effects, it is of relevance to explore the uptake of this molecule in pancreas and islet cells. Concerning oral administration, there have been few studies of intestinal digestion, uptake and further organ distribution of glycosphingolipids, including sulfatide. Among these are investigations by Nilsson *et al*., [20,21], more than three decades ago, in which glycosphingolipids (i.e. cerebroside and sphingomyelin) fed to rats were incorporated into the small intestine. However, the majority of the sphingolipids investigated were degraded within the intestine, which has also been shown by Schmelz *et al*. [22], thus releasing the backbone of ceramide and subsequently sphingoid bases, which is rapidly taken up by the intestinal cells. Sulfatide has been shown to be relatively resistant to acid hydrolysis [23,24], and therefore to have potential to be taken up as an intact molecule in the gastrointestinal tract.

To explore the uptake of exogenously added sulfatide, the following experiments were performed: *i) *uptake in the gastrointestinal tract and further transport to pancreas was studied by oral administration of radioactive sulfatide to BALB/c mice, *ii) *uptake of specific sulfatide isoforms (radioactive) by the pancreas after i.p injections in the type 2 diabetic model ob/ob mouse, *iii) *sulfatide uptake and metabolism, *in vitro*, in isolated islets of Langerhans prepared from Lewis rat was examined and different isoforms of sulfatide were compared.

## Results

### Uptake of ^3^H-sulfatide administered orally to BALB/c mice

Radioactive ^3^H-sulfatide taken up in the gastrointestinal tract was determined by scintillation counting, and radioactivity was found in isolated stomach, duodenum/jejunum, ileum and terminal ileum 1–6 hours after administration (Table 1). This finding supports the idea that sulfatide and/or its degradation products has been taken up in these compartments. Significantly lower radioactivity was found in the stomach 5–6 hours than 1–2 hours after sulfatide injection, and in the intestinal parts 24 hours as compared with 5–6 hours after administration. This indicates that sulfatide had been degraded and/or transported to another compartment by those time points. In the intestinal parts, approximately 1–5% of the orally applied sulfatide was found and in the stomach the corresponding number was 10–25% (1–2 hours after administration).

**Table 1:** Radioactivity measurements in lipid extracts of intestine, stomach and liver after oral administration of ^3^H-sulfatide

Hours after sulfatide load	Stomach dpm/mg protein	Duodenum/Jejunum dpm/mg protein	Ileum dpm/mg protein	Terminal ileum dpm/mg protein	Liver dpm/mg protein
1–2	6210 ± 1203 (8)	360 ± 58 (8)	1208 ± 238 (8)	2973 ± 2290 (3)	7 ± 2 (5)
5–6	522 ± 145 (7)**	222 ± 47 (7) (n.s.)	473 ± 115 (7)*	523 ± 93 (7) (n.s.)	15 ± 3 (7) (n.s.)
24	n.d. (3)	40 ± 10 (7)**	55 ± 8 (4)**	40 ± 10 (4)**	n.d (3)
			n.d. (3)	n.d. (3)	

BALB/c mice received sulfatide (250 nmol-1 μmol/mL, 1.7 × 10^6 ^dpm/mouse of ^3^H-sulfatide) orally, whereafter the animals were sacrificed after 1–2 hours (n = 8), 5–6 hours (n = 7) or 24 hours (n = 7). Values are mean ± SEM (n). * p < 0.05; ** p < 0.01, where 1–2 h are compared to 5–6 h after sulfatide load and 5–6 h are compared to 24 h after sulfatide load. n.d. = not detected, n.s. = not significant.

The liver, which has a rapid lipid turnover, showed low levels of radioactivity 1–6 hours after administration as compared with the intestine (Table 1), and in pancreas no radioactivity could be detected in either of the experiments. In serum, 2–3 dpm/μL sera could be detected in four out of the seven samples at 5–6 hours after sulfatide administration, whereas the rest of the time intervals were below detection limit. Faeces were only examined qualitatively, as described below, owing to the large variation of sampling between experiments.

**Figure 1:**
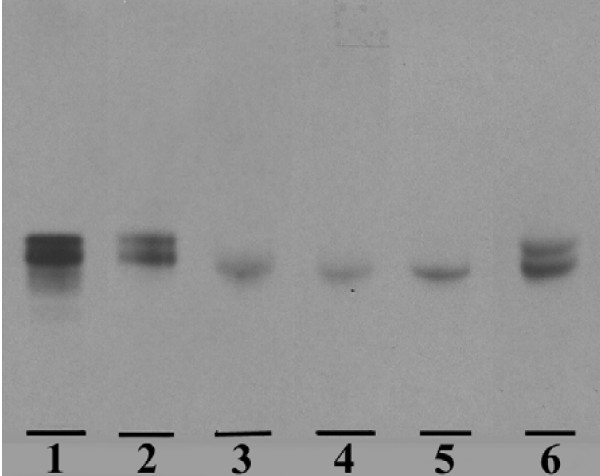
**^3^H-sulfatide uptake in the gastrointestinal tract**. The autoradiography plate is showing the uptake of ^3^H-sulfatide (brain derived) in the gastrointestinal tract isolated from BALB/c mice 1–2 hours after oral sulfatide administration. Lipid isolation, thin-layer chromatography and autoradiography are described under "*Methods"*. Aliquots of the saponified "sulfatide fractions", corresponding to 2 500–3 500 dpm, were applied to the plate (one representative plate is shown). Lane 1, ^3^H-sulfatide; ^3^H-sulfatide present in, lane 2, stomach; lane 3, duodenum/jejunum; lane 4, ileum; lane 5, terminal ileum and lane 6 faeces.

To verify that intact ^3^H-sulfatide was present in the isolated intestine and stomach, selected purified sulfatide fractions were analysed using autoradiography (1–2 hours after administration, n = 8). The presence of two sulfatide bands in the administered sulfatide fraction (Fig. 1, lane 1, brain derived sulfatide) reflects different ceramide compositions, owing to a mixture of fatty acid isoforms. In the stomach, two corresponding bands were seen and in similar proportions, while the intestinal parts only contained the slower migrating radioactive sulfatide (Figure 1). Thus, selective uptake and/or preservation of slow migrating sulfatide fatty acid isoforms were observed in the small intestine. Faeces specimens (n = 6), in agreement with findings in the stomach, had a labeled sulfatide composition resembling the given sulfatide (Fig. 1). Owing to low radioactivity in samples isolated 5–6 and 24 hours after administration, analysis of sulfatide by autoradiography was not possible.

The total sulfatide amount (endogenous pool plus exogenous uptake) was measured using TLC-ELISA in the respective tissues, and the results are summarized in Table 2. A significantly higher concentration of sulfatide was found in the stomach, duodenum/jejunum and ileum of animals 1–2 hours than 5–6 hours after sulfatide administration. This was also observed for the stomach and intestinal parts when animals were compared 1–2 hours and 24 hours after sulfatide application. These results further support an uptake of sulfatide in the gastrointestinal tract after oral administration, followed by fast (within 24 hours) degradation and/or removal of sulfatide, which could result in the endogenous "steady-state" level(s).

**Table 2:** Sulfatide in the intestine, stomach and liver after oral administration of ^3^H-sulfatide

Hours after sulfatide load	Stomach nmol/mg protein	Duodenum/Jejunum nmol/mg protein	Ileum nmol/mg protein	Terminal ileum nmol/mg protein	Liver nmol/mg protein
1–2	0,58 ± 0,14 (8)	0,09 ± 0,01 (8)	0,24 ± 0,02 (8)	0,61 ± 0,26 (3)	0,004 ± 0,001 (5)
5–6	0,18 ± 0,03 (7)*	0,04 ± 0,01 (7)*	0,08 ± 0,02 (7)**	0,08 ± 0,03 (7) (n.s.)	0,004 ± 0,001 (7) (n.s)
24	0,26 ± 0,04 (3)*	0,03 ± 0,01 (7)*	0,05 ± 0,01 (7)**	0,05 ± 0,01 (7) (n.s.)	0,006 ± 0,003 (3) (n.s.)

BALB/c mice received sulfatide (250 nmol-1 μmol/mL, 1.7 × 10^6 ^dpm/mouse of ^3^H-sulfatide) orally, whereafter the animals were sacrificed after 1–2 hours (n = 8), 5–6 hours (n = 7) or 24 hours (n = 7). Values are mean ± SEM (n). Sulfatide was quantified using TLC-ELISA.

* p < 0.05; ** p < 0.01, with respect to the sulfatide concentrations 1–2 hours after sulfatide administration. n.s. = not significant.

### Uptake of ^3^H-sulfatide isoforms administered i.p. to ob/ob mouse

Radioactive ^3^H-sulfatide isoforms (C12:0 and C16:0) taken up by pancreas 6 or 24 hours after i.p injection were determined using scintillation counting. In animals injected with C12:0 sulfatide, 1202 ± 275 dpm/mg protein (n = 4) and 1520 ± 442 dpm/mg protein (n = 4) were detected 6 and 24 hours after administration, respectively. When C16:0 sulfatide were administered, 789 ± 135 dpm/mg protein (n = 6) and 656 ± 93 dpm/mg protein (n = 6) were detected 6 and 24 hours after administration, respectively. There were no significant changes in radioactivity (cpm/mg protein) when the different isoforms or the two time periods were compared. Approximately 0.5-1% of the radioactive labeled sulfatide isoforms given to the animals were found in pancreas after 6 and 24 hours, corresponding to approximately 1 nmol sulfatide, assuming that the sulfatide molecule was still intact.

**Figure 2:**
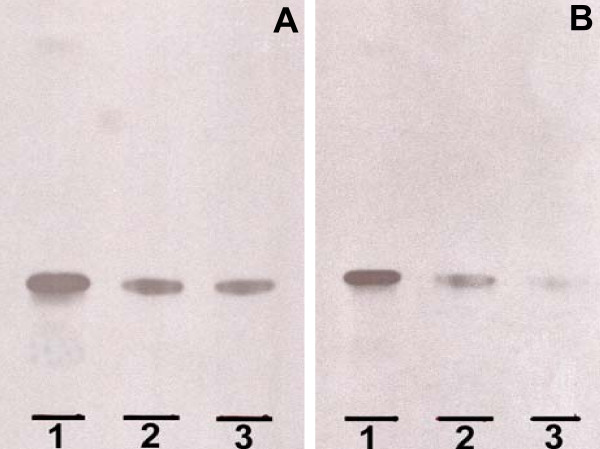
**^3^H-sulfatide uptake in the pancreas**. The autoradiography plate is showing the uptake of ^3^H-C12:0 sulfatide (A) and ^3^H-C16:0 sulfatide (B) in the pancreas isolated from ob/ob mice 6 and 24 hours after i.p. administration. Lipid isolation, thin-layer chromatography and autoradiography are described under "*Methods"*. Aliquots of the saponified "sulfatide fractions", corresponding to 2 500–3 500 dpm, were applied to the plate (one representative plate is shown). A. Lane 1, ^3^H-C12:0 sulfatide; ^3^H-C12:0 sulfatide taken up by pancreas 6 hours (lane 2) and 24 hours (lane 3) after administration. B. Lane 1, ^3^H-C16:0 sulfatide; ^3^H-C16:0 sulfatide taken up by pancreas 6 hours (lane 2) and 24 hours (lane 3) after administration.

To verify that ^3^H-sulfatide was present in isolated pancreas, saponified sulfatide fractions from pancreatic samples were analysed using autoradiography and compared with structurally characterized sulfatide isoforms (C12:0 and C16:0 sulfatide). The results show that both C12:0 and C16:0 sulfatide isoforms are taken up by the pancreas (Fig. 2).

**Figure 3:**
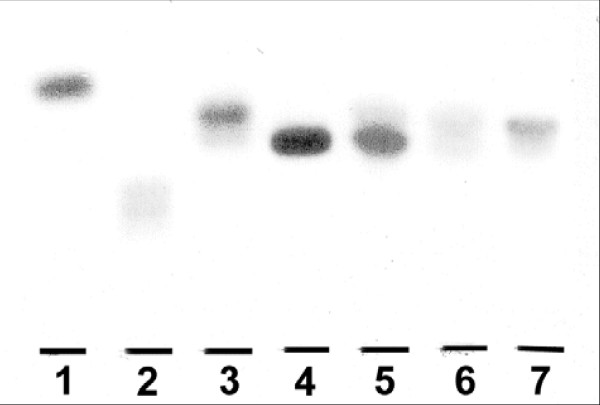
**Sulfatide in pancreas of ob/ob mice**. Thin layer chromatography of sulfatide in whole pancreas isolated from ob/ob mice given C16:0 sulfatide. The extraction and lipid isolation procedure as well as the TLC-ELISA method are described under "*Methods*". Lipids corresponding to approximately 3–4 mg tissue protein were applied (one representative plate is shown). Lane 1, seminolipid (120 pmol); lane 2, sulfated lactosylceramide (20 pmol); lane 3, sulfatide (40 pmol); lane 4, C16:0 sulfatide standard (25 pmol); lane 5, 6 hours after administration; lane 6, 24 hours after administration and lane 7, sulfatide isolated from untreated ob/ob mouse.

The total sulfatide amount (endogenous pool plus exogenous uptake) in the pancreatic tissue of ob/ob mice was examined using TLC-ELISA. Mice receiving C16:0 sulfatide showed significantly lower pancreatic sulfatide concentrations 24 hours after administration (54 ± 9 pmol/mg protein, n = 6, p < 0.05) than 6 hours after sulfatide administration (85 ± 10 pmol/mg protein, n = 6). Thus, these results lent support to the idea of sulfatide being degraded 24 hours after administration. Further support for an uptake of C16:0 sulfatide in pancreas 6 hours after i.p administration is the occurrence of a slow migrating band on the TLC plate (Figure 3, lane 5, lower band), as compared with sulfatide isolated from pancreas of ob/ob mice previously reported [3] (Fig. 3, lane 7), where mainly long-chain sulfatide isoforms were found (upper band).

Owing to the fact that the Sulph I antibody has a 5–10 fold lower affinity for C12-C14 sulfatide isoforms compared with C16-C24 isoforms (unpublished data), we were unable to obtain reliable data regarding the total amount of sulfatide (endogenous pool plus exogenous uptake) in the animals receiving C12:0 sulfatide.

### Uptake of sulfatide isoforms in isolated rat islets

**Figure 4:**
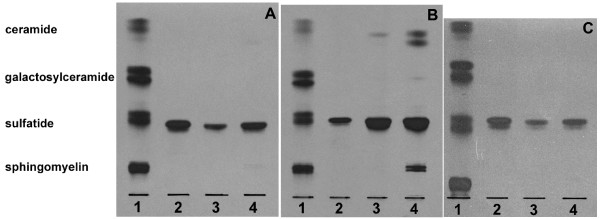
**^3^H-sulfatide uptake in isolated pancreatic islets**. The autoradiography plates are showing the uptake of ^3^H-sulfatide isoforms (C12:0, C16:0 and C24:0) in isolated rat islets of Langerhans. Isolation and culturing of islets, lipid isolation, thin-layer chromatography and autoradiography are described under "*Methods"*. Aliquots of the saponified "sulfatide fractions", corresponding to 4 000–20 000 dpm (islets cultured with C12:0 and C24:0 sulfatide) or 40 000–100 000 dpm (islets cultured with C16:0 sulfatide) were applied to the plates (one representative plate is shown). Lane 1, ^3^H-sphingomyelin, ^3^H-sulfatide, ^3^H-galactosylceramide and ^3^H-ceramide standards. A. Lane 2, ^3^H-C12:0 sulfatide standard; uptake of ^3^H-C12:0 sulfatide in rat islets after 6 hours (lane 3) and 24 hours (lane 4). B. Lane 2, ^3^H-C16:0 sulfatide standard; uptake of ^3^H-C16:0 sulfatide in rat islets after 6 hours (lane 3) and 24 hours (lane 4). C. Lane 2, ^3^H-C24:0 sulfatide standard; uptake of ^3^H-C24:0 sulfatide in rat islets after 6 hours (lane 3) and 24 hours (lane 4).

The C16:0 isoform of sulfatide was shown to be taken up by the islets of Langerhans *in vitro *(Fig. 4B, Table 3, n = 4). As early as after 6 hours of culturing, sulfatide had been degraded to ceramide and after 24 hours of culturing, sphingomyelin was synthesised from degraded or partially degraded sulfatide (Fig. 4B). Part of the radioactivity was found in the fraction migrating as standard galactosylceramide after 6 and 24 hours (Table 3), probably produced by removal of the sulfate group from sulfatide. About 0.7 ± 0.2 % (SEM, n = 7) of the ^3^H-C16:0 sulfatide added to the islets was taken up after 6–24 hours, corresponding to 0.4 ± 0.1 nmol (SEM, n = 7), which is considered a relatively large amount of sulfatide, since 500 Lewis rat islets normally contains approximately 1–2 nmol sulfatide [2].

**Table 3:** Radioactivity distribution corresponding to ^3^H-labelled lipids isolated from rat islets cultured with ^3^H-sulfatide

	C12:0 sulfatide		C16:0 sulfatide		C24:0 sulfatide	
	6 hours	24 hours	6 hours	24 hours	6 hours	24 hours
Lipids	% dpm^a^		% dpm^a^		% dpm^a^	

Ceramide	5–8	8–12	5–8	11–20	3–5	4–10
Galactosylceramide	2–4	2–4	1	2–3	1–2	1
Sulfatide	86–89	80–84	88–93	67–81	93–95	84–93
Sphingomyelin	4	5–7	1–3	6–10	1–2	1–4

^a ^% dpm = dpm for the individual scraped out lipid fractions/dpm applied to the HPTLC-plate, presented as intervals (n = 4).

The C12:0 and C24:0 sulfatide isoforms were also taken up by the rat islets, but since a lower amount of radioactivity was incorporated in these sulfatide isoforms (dpm/nmol sulfatide), as compared with C16:0 sulfatide, the degradation products could not be visualized on the TLC-plate (Fig. 4A and 4C, Table 3). A control experiment was performed by applying approximately 4000 dpm of C16:0 sulfatide (similar amount as in Fig. 4A and 4C for C12:0 and C24:0, respectively) to the TLC-plate and, as expected, the degradation products were absent (data not shown). Approximately 0.4 ± 0.1% (SEM, n = 8) of ^3^H-C12:0 sulfatide and 1.9 ± 0.6% (SEM, n = 8) of ^3^H-C24:0 sulfatide were taken up by the islets after 6–24 hours, corresponding to 0.3 ± 0.1 nmol (SEM, n = 8) and 1.0 ± 0.3 nmol (SEM, n = 8), respectively.

## Discussion

This study supports the idea that sulfatide can be taken up by pancreas when administered i.p, and *in vitro *studies further show the ability of sulfatide to be endocytosed and metabolized by pancreatic islet cells. Sulfatide can also be taken up in the gastrointestinal tract after oral administration, which includes uptake and/or preservation of selected fatty acid isoforms of sulfatide. However, with the experimental conditions used in this study, oral administration of sulfatide did not result in a distribution to the pancreatic islet cells.

The presence of sulfatide in the gastrointestinal tract of mammals has been reported in several studies [23,25-27] and the amount of sulfatide exceeded those in the kidneys, which has a high expression of sulfatide [1]. Moreover, Natomi *et al*. [23,27] suggest that long-chain hydroxylated (C22-C28) sulfatides dominate in the gastric and proximal intestinal mucosa, including duodenum and jejunum. A more recent study has also shown that the intestinal sulfatide contains short chain hydroxylated and non-hydroxylated C16:0 [28]. The band migration observed on the TLC-plate corresponds to the different sulfatide isoforms, where long chain fatty acid sulfatide migrates as an upper band and sulfatide containing short fatty acid chains and hydroxylated fatty acids (long and short fatty acid chains) migrates more slowly. Interestingly, our findings suggest selective uptake and/or preservation of slow migrating sulfatide isoforms in the intestine, corresponding to the endogenous sulfatide isoforms described above as being present in this compartment. However, the present study gives no information about the precise fatty acid composition of endocytosed sulfatide.

Oral administration of the sphingolipid sphingomyelin and simple glycosphingolipids as galactosylceramide/glucosylceramide, has shown that these compounds undergo very little cleavage in the stomach, but are instead hydrolyzed in the small intestine of mammals by alkaline sphingomyelinase, glucosylceramidase and ceramidase [21,22,29,30]. The sphingosine and/or ceramide derived from this hydrolysis have been shown to be taken up by intestinal cells and degraded to fatty acids or incorporated in complex sphingolipids [20,22]. Similar hydrolysis, involving arylsulphatase A activity, in the luminal compartment might also affect orally administered sulfatide. However, to the best of our knowledge, arylsulphatase A activity has not been reported to be present in this compartment. Sulfatide might also be desulfated by acidic hydrolysis but has been shown to be relatively resistant to acid hydrolysis, i.e. only 10% was hydrolysed at 0.1 M HCl (37°C) after 24 hours [23]. The results in this study support the idea that intact sulfatide is taken up by the intestine for degradation in the epithelial cells or for further distribution to other organs, such as the liver. However, the fact that less sulfatide is found in the intestinal compartment 6–24 h hours after injection might in addition to degradation within epithelial cells be due to that most of it has already passed thorough the intestine at those time points. Thus, the degradation of sulfatide in the intestine could be fast, such that the steady state level is determined by the passage of sulfatide through the gut. The small amount of radioactivity found in the liver fraction does not exclude the possibility that there is relatively high uptake in this compartment, since this organ has a high lipid turnover and rapid transport of these compounds.

Although our data suggest that oral administration of sulfatide does not result in further distribution to pancreas, uptake of sulfatide in the intestine might still be of interest in relation to its role in type 1 diabetes (an autoimmune disorder) for a number of reasons: 1) Sulfatide antibodies is present in type 1 diabetic individuals [31,32], indicating a possible involvement in the pathogenesis 2) sulfatide treatment delayed the onset of diabetes in BB-rats [33] 3) treatment of NOD mice with sulfatide prevented diabetes in these mice [34]. The mechanism remains enigmatic, but CD1d presentation by intestinal antigen presenting cells to functional CD1d-restricted NKT and T-cells is a promising possibility [35-37]. Thus it was an important finding that sulfatide could be taken up in the gastrointestinal tract without being fully degraded. These results imply that future studies on sulfatide treatments are feasible.

Since ob/ob mice selectively lack the C16:0 sulfatide isoform in pancreas [3], this model was used to investigate whether this specific isoform could be taken up in pancreas. This experiment was performed using i.p. injections, since oral administration of sulfatide in BALB/c mice did not show any uptake of this glycosphingolipid in pancreas. Although a low percentage of the administered sulfatide ended up in pancreas, the uptake resulted in a doubling of the sulfatide concentration in pancreas 6 hours after administration as compared with previous findings in adult ob/ob mice [3]. Thus the uptake of sulfatide seems to be high in relation to the endogenous amount. The significantly lower concentration of sulfatide in pancreas 24 hours as compared with 6 hours after administration indicates that the C16:0 sulfatide is endocytosed and thereafter partly degraded after this time period, which is in agreement with the results from the *in vitro *experiments using isolated rat islets. Thus the radioactivity remaining in pancreas 24 hours after administration probably represents intact sulfatide plus the degradation product(s) of this molecule.

Both non-native C12:0 sulfatide and the C16:0 isoform were given i.p, and similar results regarding pancreatic uptake was observed. The *in vitro *studies further support the C12:0 and C16:0 isoforms being taken up by islets, and this was also observed with the long chain beta cell sulfatide isoform (C24:0). The physical/chemical properties of sulfatide vary with different fatty acid length, including their ability to be incorporated in lipid membranes. In the present study, this phenomenon is evident in the intestine, where selected isoforms were taken up, but seem not to be the case for pancreatic islets. Thus, our results suggest that the uptake of different sulfatide isoforms in islets occurs independently of the length of the fatty acid chain, i.e. an unspecific endocytosis occurs. Sulfatide is further degraded within the islets and the production of ceramide observed probably represents a degradation product of sulfatide within the lysosomes, which is unable to exit this compartment [38]. This finding should therefore not be interpreted as increased ceramide synthesis resulting in induction of apoptosis [39].

## Conclusion

In conclusion, i.p administration of the glycosphingolipid sulfatide results in uptake in pancreas, which probably represents endocytosis by pancreatic beta cells. Although oral administration did not show pancreatic uptake, sulfatide was taken up in the gastrointestinal tract, where specific fatty acid isoforms of sulfatide are preserved. These results suggest that sulfatide could be endocytosed in relevant compartments for future *in vivo *experiments exploring the role of sulfatide in beta cell function and pathogenesis of type 2 as well as type 1 diabetes.

## Methods

### Animals

Lewis rats (male) were purchased from Møllegaard (L1., Skensved, Denmark) and BALB/c mice (male) from Bomholtgaard (Ry, Denmark) and bred in the stables at Bartholin Instituttet, Copenhagen, Denmark. Male, C57BL/6J-Lep^ob ^(ob/ob) mice were purchased from Bomholtgaard (Ry, Denmark). The principles of laboratory animal care (NIH publication no. 85–23, revised 1985), were followed, and the experiments were approved by the Danish council for animal welfare under the Ministry of Justice.

### Standards and antibodies

Sulfatide and galactosylceramide used as standard were purified from pig brain according to the procedure previously described [40]. Other lipid standards were prepared from human tissues [41]. The production and characterization of the monoclonal antibody Sulph I have been described previously [42]. The antigens recognized are the glycolipids sulfatide, sulfated lactosylceramide and seminolipid. The alkaline phosphatase conjugated anti-mouse IgG + IgM antibody was purchased from Jackson Laboratories (West Grove, PA, USA).

### Semisynthesis of sulfatide with uniform fatty acids

The synthesis of sulfatide with uniform fatty acids has previously been described [18]. Briefly, lysosulfatide was prepared from brain sulfatide, which comprises a mixture of fatty acid isoforms, using alkaline hydrolysis. The acyl chlorides of the fatty acids lauric acid (C12:0), palmitic acid (C16:0) and lignoceric acid (C24:0) were prepared by reaction with oxaloyl chloride. Lysosulfatide was reacylated with the fatty acid chloride whereafter confirmation of the sulfatide structure was performed by electrospray ionisation-mass spectrometry (Quadrupol-time of flight, Micromass, Manchester, UK) in the negative mode.

### Radiolabelling of sulfatide

Structurally characterized sulfatide, isolated from brain or synthesized with a uniform fatty acid, galactosylceramide and ceramide, used as standards, was tritium labelled in the ceramide portion with Pd(OAc)_2_/NaB^3^H_4_, as previously described [43].

### Oral administration of ^3^H-sulfatide to BALB/c mice

Phosphate buffered saline (pH 7.2) was added to tubes containing evaporated sulfatide derived from brain (mixture of isoforms, 250 nmol-1 μmol/mL), including ^3^H-sulfatide giving the specific activity of 15 000–70 000 dpm/nmol sulfatide, and the mixture was sonicated with a Branson S-250 sonifier (Branson Ultrasonics Corp., Danbury, USA) for 2 × 20 sec at 25 W prior to use. The concentration of ^3^H-sulfatide in the solution was determined by scintillation counting, and >50% was found in solution.

Male, 8–10 weeks old BALB/c mice were divided into three groups and given brain derived sulfatide orally (100 μL, approximately 20–50 nmol/mice, 1.7 × 10^6 ^dpm/mouse). The animals were sacrificed (by carbon dioxide asphyxiation) after 1–2 hours (n = 8), 5–6 hours (n = 7) or 24 hours (n = 7). Food and water were allowed *ad libitum*. From each animal, stomach, intestine, liver, pancreas and blood were dissected under sterile conditions. The intestine was divided into three parts, representing duodenum/jejunum, ileum and terminal ileum and these three parts were rinsed with approximately 2 mL phosphate buffered saline after dissection. Serum was derived from the blood samples for analysis. Tissue samples were stored at -20°C prior to analysis.

### I.p. administration of ^3^H-sulfatide isoforms to ob/ob mice

Phosphate buffered saline (pH 7.2) was added to tubes containing evaporated sulfatide with a specific fatty acid length (500 nmol/mL of C12:0 and C16:0 sulfatide), including ^3^H-sulfatide of each specific isoform giving the required specific activity of 35 000 dpm/nmol sulfatide, and the mixture was sonicated with a Branson S-250 sonifier (Branson Ultrasonics Corp.) for 2 × 20 sec at 25 W prior to use. The recovery of ^3^H-sulfatide in the solution was determined by scintillation counting and approximately 90% of C16:0 and C12:0 sulfatide were found in solution.

Male, 8–10 weeks old, ob/ob mice (blood glucose 5–15 mM) were divided into two groups and given C12:0 or C16:0 sulfatide i.p (300 μL, 5 × 10^6 ^dpm/mouse, 135 nmol/mouse). The animals were sacrificed (using carbon dioxide asphyxiation) after 6 hours (C12:0, n = 4; C16:0 n = 6) or 24 hours (C12:0, n = 4; C16:0 n = 6). Food and water were allowed *ad libitum*. From each animal, pancreas was isolated under sterile conditions and tissue samples were stored at -20°C prior to analysis.

### Culturing of islets in the presence of ^3^H-sulfatide

Pancreas form 8–10 weeks old male Lewis rats was dissected under sterile conditions and used for islet isolation. Islets of Langerhans were obtained under sterile conditions using the collagenase digestion technique described previously [44]. About 2000 islets were obtained from 3–4 Lewis rats. Islets were suspended in culture medium (RPMI 1640, Gibco, Paisley, UK, containing 11.0 mM glucose, 10% fetal calf serum and 1% penicillin-streptomycin (10,000 IU/mL-10,000 μg/mL, Gibco) and with the pH adjusted to 7.35) and incubated for 20 hours in an atmosphere of 95% air-5% CO_2 _at 37°C before addition of sulfatide into the culture medium.

Evaporated sulfatide was resolved in fresh culture medium (RPMI 1640, Gibco) by sonication (Branson S-250 sonifier, Branson Ultrasonics Corp.) for 2 × 20 sec at 25 W prior to use. The recovery of ^3^H-sulfatide in the solution was determined by scintillation counting and approximately 80–90% of the C12:0, C16:0 and C24:0 sulfatide isoforms were found in the culture medium. The concentration was found to be approximately 12–15 nmol sulfatide/mL culture medium of the respective isoform, including ^3^H-C12:0 sulfatide, ^3^H-C16:0 sulfatide and ^3^H-C24:0 sulfatide giving the required specific activity of 50 000, 1.2 × 10^6 ^and 12 500 dpm/nmol sulfatide, respectively.

Islets were divided into 500 islets/culture dish, put into culturing media (as described above) containing the separate sulfatide isoforms and cultured for 6 hours (n = 4) or 24 hours (n = 4) in an atmosphere of 95% air-5% CO_2 _at 37°C. During this period of culturing, heat inactivated fetal calf serum was used (56°C for 45 minutes). Islets were harvested and washed in phosphate buffered saline by centrifugation and stored at -20°C prior to analysis.

### Analysis of sulfatide from tissue/islets

Lipids were extracted from homogenates of tissues/islets, after protein determination (BCA Protein Assay Reagent method, Pierce, Rockford, USA), by adding methanol and chloroform to give a final ratio of chloroform/methanol/water (C/M/W, 4:8:3, by vol.) [45]. An aliquot of the lipid homogenates was mixed with 10 mL scintillation liquid (Ultima Gold, Packard BioScience, Groningen, The Netherlands) and analysed using scintillation counting (Tri-Carb 1500, Packard) to determine the specific radioactivity of ^3^H-sulfatide taken up by the tissue. Approximately 50 μL of the serum samples were analysed by scintillation counting. Further purification was performed by silica gel-60 chromatography (Merck, Darmstadt, Germany), as previously described [10], where sulfatide, phospholipids, sphingomyelin and ceramide were eluted with C/M/W (65:25:4, by vol.) and gangliosides with C/M/W (30:60:20, by vol.) The "sulfatide fraction" was saponified in methanol:1 M KOH (1:1 v/v) [3] before autoradiography or quantification using thin-layer chromatography-enzyme linked immuno-sorbent assay (TLC-ELISA), as described below.

### Autoradiography

Aliquots of the saponified "sulfatide fractions", corresponding to 2 500–3 500 dpm (tissue), 3 500–8 500 dpm (islets cultured with C12:0 and C24:0 sulfatide) or 35 000–100 000 dpm (islets cultured with C16:0 sulfatide) were applied as 8 mm bands to alumina-backed high performance-TLC plates (Merck) and chromatographed in C/M/W (65:25:4, by vol.). The plate was air dried, sprayed with Enhance spray (NEN™ Life Science Products, Zaventem, Belgium) and exposed to x-ray film for 7–14 days. The individual sulfatide bands after chromatography were identified by comparing with the migration of structurally identified sulfatide standard(s). Autoradiography performed on the "sulfatide fraction" purified from islets also included structurally identified standards of galactosylceramide, sphingomyelin and ceramide. In this experiment, liquid scintillation counting, as described above, was performed on samples after saponification and on scraped out fractions of the individual lipid bands after chromatography.

### Quantitative determination of sulfatide by TLC-ELISA

Sulfatide was identified and quantified using a TLC-ELISA method previously described [46] using the Sulph I antibody [42]. Briefly, purified standards of sulfatide and aliquots of the "sulfatide fraction" were applied as 5 mm lanes to TLC plates (10 × 20 plastic-backed, Merck) and chromatographed in C/M/W (65:25:4, by vol.). Plates were sequentially incubated with the monoclonal Sulph I antibody, alkaline-phosphatase-conjugated anti-mouse antibody and 5'-bromo-4'chloro-3'indolylphosphate. All steps were performed at room temperature. The intensity of the developed color was determined by densitometric scanning at 620 nm.

### Statistics

The sulfatide content and/or uptake in tissues and islets were presented as mean values ± SEM. The significance of difference was evaluated by using Student's t-test (significance level set at < 0.05).

## Competing interests

The author(s) declare that they have no competing interests.

## Authors' contributions

MB carried out part of the lipid analyses and collected the data, participated in the design of the experiments and drafted the manuscript. TO carried out the administration of sulfatide to mice. J-E M and KB participated in interpretation of data and were involved in revising the manuscript critically. PF made substantial contribution to the design of the study, interpretation of data and in revising the manuscript critically. All authors read and approved the final manuscript.
